# Santiago Ramón y Cajal and Ivan Petrovic Pavlov: their parallel scientific lives, schools and nobel prizes

**DOI:** 10.3389/fnana.2015.00073

**Published:** 2015-06-02

**Authors:** Jairo A. Rozo, Antonio Rodríguez-Moreno

**Affiliations:** Department of Physiology, Anatomy and Cellular Biology, University Pablo de OlavideSeville, Spain

**Keywords:** Cajal, Pavlov, nobel prize, madrid congress 1903, city of Moscow prize, history of neuroscience, teaching

## Abstract

Santiago Ramón y Cajal was not only a great scientist but he was also a dedicated teacher who managed to create his own School in Spain. Cajal was active at the end of the XIX and the beginning of the XX century, a period in which Ivan Petrovich Pavlov, another great contemporary scientist, also established a strong School in Russia. While these two acclaimed scientists shared a similar vision on science, a view they also conveyed to their disciples, they applied quite distinct criteria in the way they dealt with their followers. Interestingly, despite the geographic and idiomatic barriers that had to be overcome, the paths of these two great figures of XX century science crossed at least three times. First when they competed for the City of Moscow Prize, second when they both attended the “Congreso Internacional de Medicina de Madrid” (Medicine International Congress in Madrid) in 1903 and finally, they competed on four consecutive occasions for the Nobel Prize in Physiology or Medicine. Here we discuss their scientific vision, their different attitudes in the interaction with disciples and the distinct circumstances in which their paths crossed.

## Introduction

**Figure 1:**
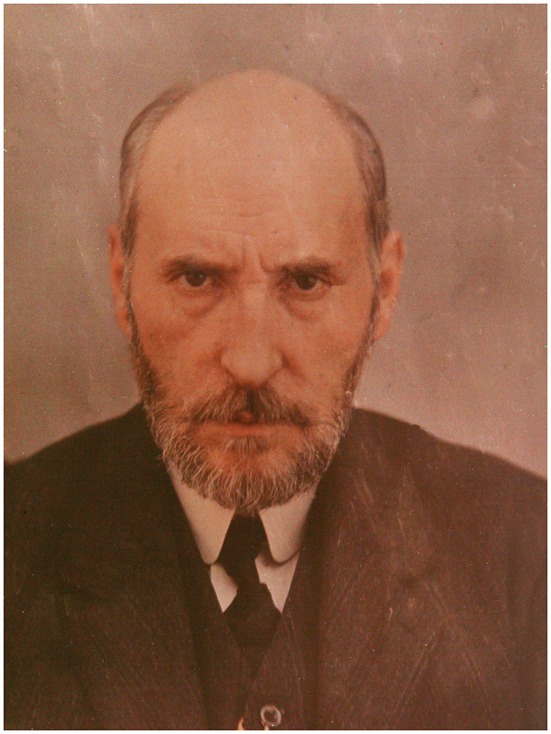
**Santiago Ramón y Cajal**. Cajal in a photograph from approximately 1906 when he was awarded the Nobel Prize in Physiology or Medicine (image reproduced with permission from the Cajal Institute, Madrid).

**Figure 2:**
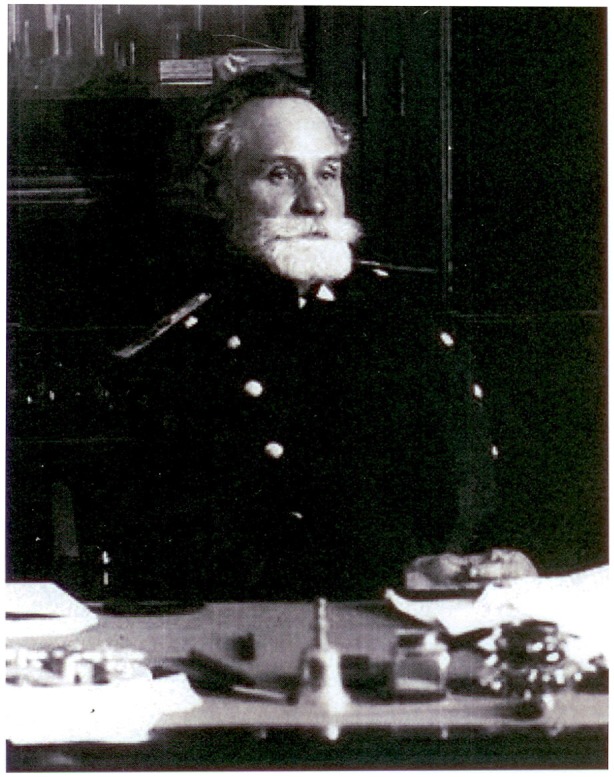
**Ivan Petrovich Pavlov**. Pavlov at his desk in the Imperial Military Medical Academy, St Petersburg in 1904, when he was awarded the Nobel Prize in Physiology or Medicine (image reproduced with permission from The Institute of Experimental Medicine of the Northwest Branch of the Russian Academy of Medical Sciences, St. Petersburg).

Santiago Ramón y Cajal (1852–1934; Figure 1) and Ivan Petrovich Pavlov (1949–1936; Figure 2) are two important figures in science who were not only contemporary, but they also both had to overcome difficult conditions to carry out science in their respective countries. Nevertheless, both became prominent national and worldwide figures, gaining the highest recognition with the award of the Nobel Prize in Physiology or Medicine, and becoming models for their fellow scientists in Spain and Russia, respectively.

Cajal and Pavlov were born less than 3 years apart, they received the Nobel Prize in Physiology or Medicine within 2 years of one another, and they died within a 2 year interval. Although they had quite distinct personalities, their lives had some similarities, as an impulse to science in their homelands during the same period (Table 1). Their lives were both characterized by their tremendously disciplined willingness to work, a common vision of their work as scientists, a great concern for the future of science in their respective countries, and a strong commitment to young researchers and their disciples.

**Table 1:** **The lives of Cajal and Pavlov, their career milestones**.

Year	Santiago Ramón y Cajal	Ivan Petrovich Pavlov
1849		Ivan Petrovich Pavlov, born on the 26th of September in Ryazan (Russia).
1852	Santiago Ramón y Cajal, born on the 1st of May in Petilla de Aragón (Spain).	
1875	Becomes Assistant in Anatomy at The School of Medicine in Zaragoza.	Becomes Doctor in Natural Sciences
1876	Obtains a permanent position as Assistant at the Hospital of Zaragoza.	Becomes Technician and Assistant at the Veterinary Institute of St. Petersburg.
1877	Becomes Doctor in Medicine.	Develops a new pancreatic fistula procedure.
1879	Obtains a permanent position as Director of the Anatomy Museums in Zaragoza	Graduates in Medicine.
1883	Becomes Full Professor at the University of Valencia.	He becomes Doctor in Medicine.
1884	He starts to publish in fascicles the *Manual de Histología*.	Is designated Assistant Professor at the Military Medical Academy of St. Petersburg.
1886		Is named Director of the Botkin laboratory.
1887	Cajal is introduced to the Golgi technique.	He works at the Botkin laboratory.
1888	Develops a *double impregnation procedure*. He postulates the autonomy of the neuron.	
1889	He attends the German Anatomical Society Congress. Kölliker supports him.	
1890	Cajal studies the embryonic development of the nervous system.	He becomes Full Professor at the Military Medical Academy of St. Petersburg.
1891	He presents for first time the law of dynamic polarization of neurons in a Congress held in Valencia.	Is designated Director of the Department of Physiology at the Imperial Institute of Experimental Medicine in St Petersburg.
1892	Is awarded a Full Professorship position at the Central University of Madrid.	
1895		Is designated Full Professor in Physiology at the Military Medical Academy of St. Petersburg.
1897	He begins the publication of his great treatise *Texture of the nervous system of man and the vertebrates* in fascicles.	Publishes his book *The work of digestive glands*.
1900	He was awarded the City of Moscow Prize.	
1903	Cajal participates in the XIV International Congress of Medicine in Madrid.	Pavlov participates in the XIV International Congress of Medicine in Madrid.
1904	Cajal finishes the book *Texture of the nervous system of man and the vertebrates*	Receives the Nobel Prize in Physiology or Medicine for his work on digestive physiology.
1905	The Science Academy of Berlin concedes the Helmholtz Gold Medal to Cajal.	The method of “artificial” conditional reflexes is introduced into his laboratory.
1906	Receives the Nobel Prize in Physiology or Medicine for his contributions to the knowledge of the nervous system.	
1911	*Histologie du système nerveux de l’Homme et des vertebrés* appears in French.	Pavlov begins extensive studies related to cortical inhibition.
1923		Publishes his book, *Twenty Years Experience in Objective Study of Higher Nervous Activity (Behaviour) of Animals*
1926	Inauguration of the Institute Cajal for Biological Research.	
1927		Publishes his book *Lectures on the Work of Large Hemispheres of the Brain*
1933	Publishes *Neuronism or reticularism*	
1934	Ramón y Cajal dies in Madrid at 22:45 on the 17th of October. He was 82 years old.	
1936		Pavlov dies in Saint Petersburg on the 27th of February. He was 86 years old.

## Cajal’s and Pavlov’s Vision of Science

Cajal’s and Pavlov’s vision of science was inherited from *positivist epistemology*, *scientific monism*, the *theory of evolution* and from *experimental methodology*. From this position, Cajal encouraged the adoption of science-based medicine in Spain, moving away from the *vitalism*, *metaphysics*, *dualism*, *and empiricism* that dominated much of the XIX century. It should be remembered that most medicine at the time was dominated by *vitalism* and *empiricism*. However, thanks to the new experimentalist approach it began to be considered that disease is not a defensive act of the “vital principle” but rather a consequence of a malaise of cells in the body, and that experimentation is the best way to overcome clinical empiricism.

Cajal and Pavlov rejected *vitalism* and *metaphysics* as a way of attaining knowledge, and they advocated the application of the scientific method. They conceived that all activities of the organism had physical basis. Against *dualism*, they defended the principle of *scientific monism*, which holds that mental activity could be reduced and explained by the physiology of the nervous system.

In agreement with *evolutionism*, Cajal and Pavlov based their ideas on the existence of a continuum in phylogenetic evolution that allowed them to perform studies on lower species and to some extent extrapolate the results to higher species difficult or impossible to investigate in the laboratory. Obviously, it was a true breakthrough for Cajal to be able to use techniques that stained the nervous cells in lower species and at early stages of the development. The nervous system of these animals is relatively simple, which implied that he could find principles applicable to the nervous system of different species, and even to humans.

Cajal and Pavlov considered that the prestige of a scientist depends on the original facts he produces. “Hypotheses come and go but the facts remain. Theories abandon us, whereas the facts defend us” (Ramón y Cajal, 1940). Cajal was always aware of the commitment that scientific work implies, writing: “… people insist little in a form of attention that could be called *brain polarization* or *chronic attention*, where all our powers are tirelessly focused on an object of study for months and even years” (Ramón y Cajal, 1940). Indeed, Cajal always advocated that overwork and excessive attention creates talent, as opposed to the widespread notion that it surges from thin air. Cajal did not regard himself as a genius but rather a stubborn workhorse of Spanish science.

Similarly, Pavlov in his letter to the Soviet youth, speaks about the quality he defined “passion for science”: “The most important, I insist, is the intense and persistent concentration of the mind: the aptitude to think incessantly about a certain matter, to go to bed and to get up thinking about it constantly” (Frolov, 1937).

Despite their similarities, as stated above, Pavlov’s view on how a School should be managed contrasted considerably from that of Cajal (Blanco, 2014).

## The Attitudes of Cajal and Pavlov Towards their Schools

Both Pavlov and Cajal can be considered as researchers who reached the category of maestro. Each of them achieved undisputed recognition in his area and, additionally, both developed new techniques. The technique developed by Pavlov was the preparation of the fistula from the parotid or submandibular gland that he used to study learning by conditioning. Cajal modified and improved the chromium silver technique developed by Golgi to stain nerve cells, and devised other novel staining methods (reduced silver nitrate, formalin-, uranium or sublimed gold). These techniques allowed him and his disciples to obtain new data and develop novel theories, eventually forming a School of followers attracted by Cajal’s reputation and the new techniques he had developed.

Two types of collaboration emerge within a School: direct and indirect (De Castro, 1952). Direct contributions are those that follow the lines identified by the maestro and are carried out under his supervision, without deviating from the pattern or main idea. In this case, the laboratory work will carry the hallmark of the school, which applies equally to all involved, and the laboratory and its work revolve around the maestro. By contrast, the indirect collaborator is much freer. He/she selects the topic to investigate irrespective of the desires of the maestro. In this case, the School’s work will be distinguished by its originality and by the author’s own personality. Here the laboratory’s role varies and the investigator can follow different paths, parallel or not to those of the maestro.

Pavlov’s school represents a clear example of direct cooperation between the disciples and the master. He was the head of the laboratory, he guided and planned the work of his many followers, intervening in their work if necessary, and he organized and selected the results of interest. Indeed, he evaluated all the individual experiments and he evaluated the inter-related approaches to understand the issues in question. Since Pavlov was the head and his disciples the hands, the basic ideas came from him and therefore, any intellectual property generated through the huge amount of data collected by his different disciples belonged to him.

Pavlov says in the preface to his book *The work of the digestive glands* (Pavlov, 1902) “I use the word “we” to indicate the entire laboratory. We always name the researcher when describing the experiments but the object of the experiment, its significance and its relationship to the series of experiments carried out is considered from the perspective of the laboratory, as opposed to any isolated opinion or the sole findings of the individual researcher. This way of working has a specific advantage for the reader, as it allows him to really ascertain how the findings were made, given that they are derived from a consistent line of study and shaped through harmonious experimentation” (De Castro, 1952).

Pavlov had 80 disciples during the first period of his scientific activity, which was focused on digestion (pre-1904). Over the years of his studies into higher nervous activity he mentored about 200 disciples, not counting foreign visiting students (Frolov, 1937). Among these, V. N. Massen, a gynecologist who established the initial aseptic and antiseptic procedures in the laboratory, has been highlighted as one of the most constant aides of Pavlov (Todes, 2002). N. I. Damaskin and E. A. Ganike (biochemists), and A. P. Sokolov (a histologist), also made important contributions. Ganike held a prominent place in the laboratory and he was considered as Pavlov’s right hand man from 1894 until his death. He handled the finances and he oversaw various activities, and it was he who prepared the annual activity reports that were approved by Pavlov. Another of Pavlov’s important disciples was Nikolai Kharitonov, his surgical assistant in whose absence Pavlov said he had lost his hands.

Pavlov’s ideas spread internationally thanks to his disciples. For example, Gleb von Anrep, spread the word when working at the University of London and of Cambridge after 1920, and for 20 years when acting as the director of the Department of Physiology of the Egyptian University of Cairo. Boris P. Babkin, who introduced the ideas of Pavlov into England and Canada, was a member of the Canadian Royal Society. William N. Boldyreff immigrated to Japan in 1918 and in 1922 he went to the USA, where he became director of the Pavlovian Laboratory and Hospital in the state of Michigan until 1940. In Poland, Jerzy Konorsky developed Neuropsychology and William Gantt, who worked with Pavlov between 1925 and 1929, played an important role in developing the ideas of Pavlov in the USA (Klimenko and Golikov, 2003).

**Figure 3:**
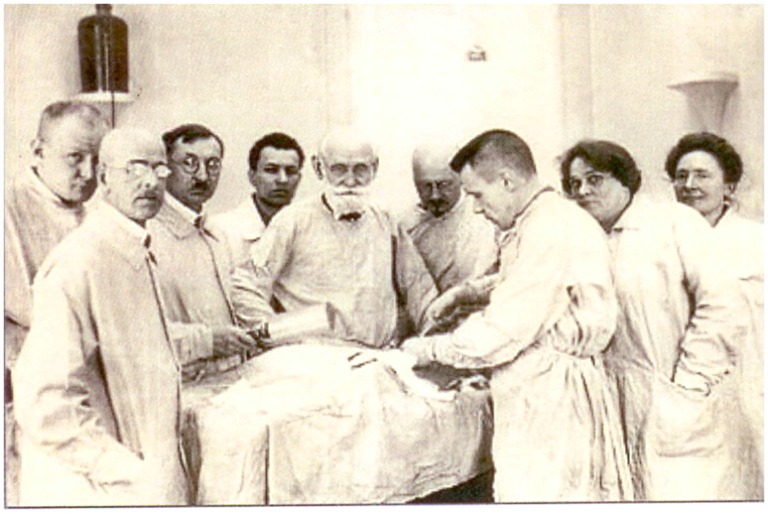
**Pavlov with three colleagues and disciples operating on a dog in the Physiology Department, Imperial Institute of Experimental Medicine, St Petersburg**. Second on the left: G. von Anrep, operating: Alexander Speranskii (image reproduced with permission from The Institute of Experimental Medicine of the NorthWest Branch of the Russian Academy of Medical Sciences, St. Petersburg).

How Pavlov organized the laboratory resembled the production process in a factory. He was a strict manager who put his new disciples to the test while they were being trained, prior to offering them any research work. Ideally, an initial training was offered for the work that Pavlov had in mind in order to bring new members of the laboratory up to the level of “expert hands”. The experiments they carried out and their results would have otherwise been considered useless. Once the best co-workers had been identified, he engaged them in research topics he considered most important. In the laboratory, Pavlov demanded timeliness, accuracy and quality of work. This research machinery or factory was essentially held together thanks to Pavlov’s own personal qualities, his leadership, his energy and inspiration, as well as his exceptional organizational skills (Todes, 2000, 2002; Figure 3).

Conversely, Cajal’s School was characterized by indirect collaborations in which intellectual freedom prevailed. Nevertheless, the characteristic features of Cajal’s personality could always be detected in his disciples’ work and their individual efforts helped consolidate his own research. This became especially relevant when their research objectives led them along paths distinct to those chosen by their maestro. Cajal never presented opposition to his followers expressing and developing their own independent ideas, that rather, he welcomed and solicited. Cajal did not want devotees of just a single book and followers of a single master. In his own words: “My aim is to offer support and to illuminate the way, fully respecting individual initiatives” (De Castro, 1952).

Cajal believed that an ambitious scientist should remain undisturbed during the training period because the available time was limited and had to be devoted to individual work. He was aware that for young scientists to be successful they must dedicate all their available energy to their work. As such, a young researcher should not establish a School, which would represent a distraction and absorb too much energy at a time when they had not gained sufficient experience (De Castro, 1952). For Cajal, teaching is a job for the wise and to maximize the benefits to be gained from them, efforts must be made to spread their ideas as widely as possible, guaranteeing the well being of the nation. The creation of a school is vital to the most successful researcher (De Castro, 1952).

Cajal’s School truly emerged in the early XX century when the Spanish state began to support him, providing him a well equipped laboratory and founding the “Laboratory for Biological Research”. This progress followed the award of the Moscow Prize in 1900 at the International Congress of Medicine held in Paris. In previous years, Cajal had had young assistants who helped him with his laboratory work. For example, De Castro refers to Claudio Sala, Carlos Calleja, Isidoro Lavilla, Tomás Blanes, Federico Olóriz-Ortega, and Pedro Ramón y Cajal (Cajal’s brother), although the authentic and first true disciples of the Master were Jorge Francisco Tello and Domingo Sánchez. The first original works coming from Cajal’s disciples were produced by Tello and they first appeared in their laboratory journal. Nicolás Achúcarro, who trained with Luis Simarro (who first introduced Cajal to Golgi and silver nitrate methods) and Alzheimer, was a subsequent addition to this list, followed by Pío Del Río-Hortega, Gonzalo Rodríguez-Lafora, and José Maria Villaverde. The last of the direct disciples, who began working with Cajal between 1902 and 1916, were Rafael Lorente de Nó and Fernando De Castro, completing this first generation.

The generation mentioned above, trained in turn new disciples who gave rise to a second generation. Between 1916 and the outbreak of the Spanish Civil War, the School grew to the sum of 41 disciples, especially those working with Tello, De Castro and Del Rio-Hortega. Unfortunately, the onset of the Civil War led to a dispersion of the School, many of the disciples going into exile and the few who remained in the country living and worked in very difficult conditions (Aguirre, 2002).

Cajal’s believed a teacher should guide his disciples, identifying adequate lines of research, steering them through the literature, and helping them acquire the necessary knowledge and skills (languages, artwork, writing, etc …). The teacher must gradually test the student’s capacities, proposing accessible research topics derived from his/her basic interests. Once they have developed sufficient technical and speculative capacity, they will gradually face more stimulating research challenges (Ramón y Cajal, 1940).

To understand Cajal’s vision, it is perhaps best to consider how he describes this in his own words: “When the noble investigator can work by himself, take care to imbue in him a pleasure for originality and allow him to suggest new ideas, even when they do not conform with the theories of the School. The true glory of the maestro is not to train his disciples to follow him but rather, to instill the wisdom that will make them capable of surpassing him. The truest ideal should be to create new unique spirits where possible, in order to drive the machine of progress. The generation of docile and indistinguishable followers indicates that the master has been more centered on himself than in furthering Science, and benefiting his country” (Ramón y Cajal, 1940, Figure 4).

**Figure 4:**
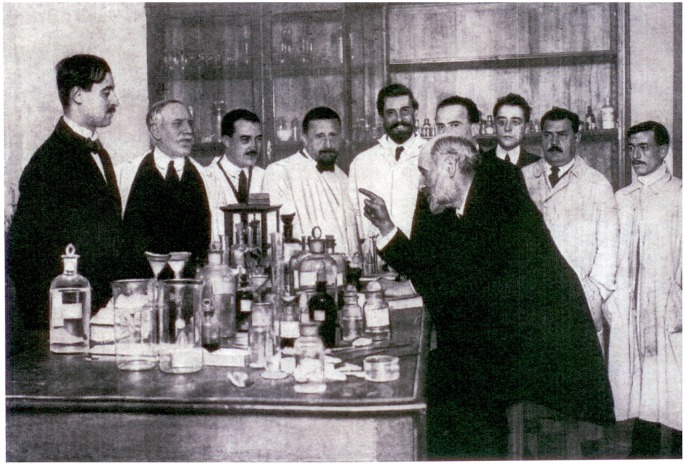
**Cajal with some of his disciples in his laboratory**. First and second on the left: G. Lafora and D. Sánchez, respectively, in the middle N. Achúcarro (Courtesy of the Cajal Institute, Madrid).

Cajal, like Pavlov, embodied many of the qualities required for scientific success: an indefatigable capacity for work; a capacity to describe observations; a patience bordering on obstinacy to develop new methods; a dexterity and skill to replace expensive experimental set-ups with simple custom-made pieces of equipment; a continuity and indefatigable zeal to obtain facts; and above all, a flexibility to change or revise opinion and to correct errors. Cajal tried to convey these qualities to his disciples in a motivating environment, offering total freedom. Thus Pavlov and Cajal shared a similar vision of how science should be done, a view that they conveyed to their disciples (Ramón y Cajal, 1940; Asratian, 1949).

## Did Cajal and Pavlov Ever Meet?

Given that we are considering two contemporary figures, one might expect that they would have maintained some contact, at least by correspondence. For example, Cushing (the father of modern neurosurgery) and Pavlov communicated by letter and they also met in person. Cushing also established indirect contact with Cajal through his disciple Penfield, who was also a disciple of Del Río-Hortega, a member of Cajal’s school. Indeed, Penfield was the first American scientist who worked with Cajal’s group (Aguirre, 2002; Zamora-Berridi et al., 2011).

**Figure 5:**
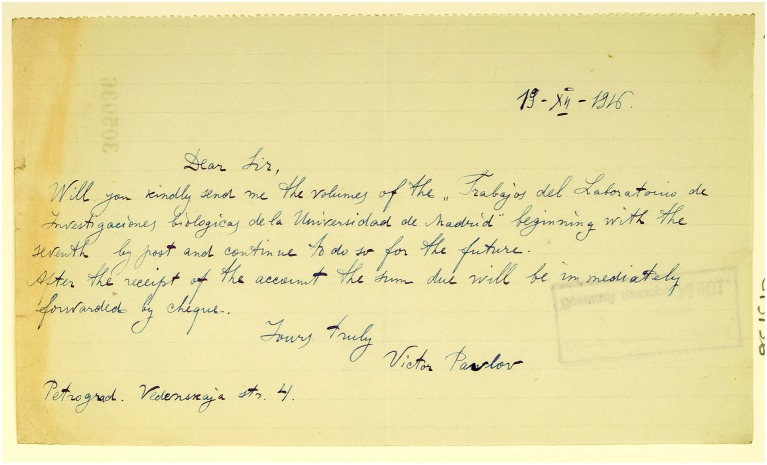
**Letter from the “Legado Cajal” from Victor Pavlov to Cajal in 1916 (Cajal Institute, CSIC, Madrid)**.

There are no records of letters exchanged between these two scientists, neither at the Cajal Institute in Madrid (part of the CSIC—the Spanish National Research Council—and where the “Cajal’s Legacy” is located) nor at St Petersburg branch of the Archive of the Russian Academy of Sciences (with holds Pavlov’s papers). Only one letter addressed to Cajal from Victor Pavlov in 1916 has been found at the Cajal Institute, requesting copies of the journal edited by Cajal (Figure 5). Unfortunately, at present it is not possible to confirm whether or not this letter is really from Ivan Pavlov as it is not signed by him but rather by his son Victor Pavlov. Victor was a promising student but it was his daughter Vera who later joined Pavlov to perform research on conditioned reflexes (Todes, 2000). Therefore, it is uncertain whether the letter came from Ivan Pavlov’s son or even from a completely different person. Indeed, one of Pavlov’s biographers, Todes, who had direct access to various files while working in St. Petersburg, found no record of any written contact with Cajal. According to him, Pavlov must have known about Cajal’s research but there are no records of discussions between them, possibly because of their different expertise and to language problems (the only language they had in common was German).

Todes also notes that the only mention of Cajal found in Pavlov’s archives was a letter of December 1924 from Cushing to Pavlov. In this letter, Cushing tried to persuade Pavlov to write a book in English on conditional reflexes: “I only regret that we do not have access to your writings in English. I’m afraid we may have to learn Russian in the same way we have had to learn Spanish to follow the studies of Ramon y Cajal in Madrid” (Archive of the Russian Academy of Sciences, quoted in Zamora-Berridi et al., 2011).

Alternatively, contacts between Pavlov and Cajal could have been mediated through their disciples, although there is no evidence for this. Therefore these two scientists might have met only once, at the international meeting in Madrid in 1903.

## Congress in Madrid, 1903

Cajal was not only a speaker, but he was also a member of the Organizing Committee of the XIV International Congress of Medicine held in Madrid in 1903, where Pavlov also offered a lecture (Campos-Bueno, 2003; García-Albea Ristol and García-Albea Martín, 2010; Campos-Bueno and Martín-Araguz, 2012). The XIV International Congress of Medicine in April 1903 marked an important milestone for Psychology and Neuroscience, and for Science in general. As Campos-Bueno indicated, two discoveries that would mark a century in the study of the brain and behavior were unveiled before the public. “Both works considered the body as a whole and as such, both these methods had been developed *in vivo*, overcoming the limitations of classical Anatomy and Physiology. We refer to the works of Ivan Petrovich Pavlov and Santiago Ramon y Cajal” (Campos-Bueno, 2006).

Two of the four papers Cajal presented at the meeting are especially interesting: the first because it was presented just before Pavlov’s and the second because it is transcendental to the defense of the neuron doctrine. Thus, it would appear highly likely that Cajal and Pavlov met on April 28th, 1903 (García-Albea Ristol and García-Albea Martín, 2010). Cajal’s presentation was titled “*Plan of structure of the optic thalamus*” in which the mapping of the thalamus was thoroughly discussed for the first time, acting as a point of transit for the sensory pathways and other projections (Campos-Bueno and Martín-Araguz, 2012). Interestingly, after Cajal’s presentation, Ivan Pavlov presented the world’s initial discoveries on *Psychical Secretion*, later known as *Conditional Reflexes*. Since they presented their respective papers in the same auditorium, in the same event and on the same day, one preceding the other, and being two of the most distinguished scientific figures of the day, it is more than likely that they were formally introduced there. However, we have found no photographic or written record to confirm that such an encounter did in fact take place.

The talk delivered by Pavlov was titled “*Psychology and Experimental Psychopathology in animals*”, in which he surprised the audience by presenting a series of new facts obtained through his research on the digestive glands (research that would lead him to obtain the Nobel Prize in the following year). Indeed, these studies laid the foundation for him to plan the following years of his research, from then on focusing on the nervous system and the animal’s ability to learn in the midst of a changing environment (Pavlov, 1927, 1955). Thus, this paper contained the seed of future works and marked a turning point for Psychology, providing a scientific method to study learning and memory processes in higher mammals.

Cajal’s lecture that followed was titled “*Critical considerations on the theory of A. Bethe on the structure and connections of nerve cells*”. Cajal was already the leading figure of Spanish science at that time and he was engaged in a theoretical struggle with European reticularist scientists, given that he and other colleagues supported and defended the *neuron theory*. This hypothesis postulated that the nervous system was composed of independent, autonomous cells that communicated with other cells by contiguity rather than continuity, as argued by reticularists. As Cajal hinted (1923), the character of this speech was more controversial as its purpose was to stimulate a debate on the reticularist theories of Bethe, in order to propose, promote and discuss the important issue of neuronal connections, and the fine structure of the nervous protoplasm. The importance of this paper is that it marked the beginning of a series of studies by Cajal, which would lead to a new method of histological staining and the confirmation of the neuron doctrine, as well as opening a line of research on degeneration and regeneration in the nervous system.

Simarro was present when Cajal delivered his speech and he also presented data obtained through his new technique to stain the neurofibrillary network (later modified by Cajal and that would ultimately become the reduced silver nitrate method), which demonstrated that their inner neurofibrils did not form part of an neuronal network as argued by the reticularists. An important feature of Simarro’s method was that it allowed the cell to be studied as single entity. Interestingly, this staining was performed on a live animal before it was sacrificed, thus avoiding potential *post mortem* artifacts (Ramón y Cajal, 1923; Campos-Bueno, 2006; Frixione, 2009).

The studies of Cajal and Pavlov had some common ground. They both focused on the organism as a whole, overcoming the constraints of classical anatomy and, as previously indicated, they both developed innovative experimental techniques: Pavlov developed the permanent fistula, which allowed salivation to be measured in living, healthy dogs; Cajal developed new methods to stain neurons before the animals were sacrificed from the technique devised by Simarro. Both discoveries stressed the importance of cell contiguity as a functional feature of the brain. Finally, as a crucial element in their theories, they were both trying to find a basic unit for an objective investigation of mental activity (Campos-Bueno and Martín-Araguz, 2012). Pavlov presented reflexes as basic behavioral units thanks to his theory of conditional reflexes, while Cajal studied the nerve cell as the basic unit of the nervous system (Campos-Bueno, 2006).

## The Moscow Prize

The Moscow Prize was the first time that Cajal and Pavlov were contenders for an academic award. Tsar Nicolas II established the award in August 1897, during the Russian convention of the XII International Congress of Medicine. The prize was to be awarded to the most original research submitted to subsequent medical congresses and its first recipient was presented with the award 3 years later in Paris. Pavlov attended the Paris meeting and was keen to be awarded this prize (Campos-Bueno and Martín-Araguz, 2012).

The Paris meeting was an important moment in the academic life of Pavlov and Cajal, since both competed for the Moscow Prize. However, they did not have the opportunity to meet there in person as Cajal could not attend the Paris congress in August 1900 due to health problems. The third scholar nominated to compete with Cajal and Pavlov was Metschnikoff. Cajal won the first edition of the award, obtaining 14 votes in favor as opposed to 6 obtained by Metschnikoff and 3 by Pavlov. At the same meeting, it was also decided that the following congress would be held in Madrid, in 1903 (Campos-Bueno and Martín-Araguz, 2012). The Spanish people rejoiced with this decision as, to some extent, it compensated for the recent military defeats, and as Cannon (1949) said: “Choosing Cajal was considered a kind of racial triumph”.

The Moscow Prize was also very important for the academic life of Cajal, producing recognition by the Spanish government. He received the Great Cross of the Order of Isabel the Catholic, the Great Cross of Alfonso XII and he was appointed Counselor of Public Education. In the same year, 1900, he was also appointed Director of the “Alfonso XIII” National Institute of Hygiene (Cannon, 1949) and the government approved the establishment of a laboratory for Cajal, the “Laboratory of Biological Research” (De Carlos and Borrell, 2007).

Later, Cajal and Pavlov were to compete again, this time for the Nobel Prize in Physiology or Medicine. They were nominated together on 4 consecutive occasions (1901–1904), with Pavlov finally being awarded the Prize in 1904 and Cajal sharing it with Golgi in 1906.

## Pavlov and the Nobel Prize

As mentioned above, Pavlov was nominated for the Nobel Prize in Physiology or Medicine for four consecutive years (1901–1904) and every time the Committee was faced with the question: “To what extent are the works from Pavlov’s laboratory actually his own?” This question arose for good reason. At different lectures, Pavlov had openly acknowledged the collective efforts of the whole laboratory and he named several colleagues who had performed experiments on which his presentations were based. Therefore, the Committee did not know if Pavlov results were indeed original contributions or merely a compilation of the contributions of his colleagues. In the early XX century, the idea persisted that science was created by great minds and not by a machine or scientific apparatus as those in Pavlov’s laboratory. The Nobel Committee finally decided that the work from Pavlov’s laboratory was truly his merit even taking into account his scientific profile and his way of organizing the laboratory work (Todes, 2002). To assess the work of Pavlov, in 1901 the Nobel Committee carried out an evaluation entrusted to two eminent physiologists of the time, Johansson and Tigersted. They visited St. Petersburg on June 8th (1901) to witness Pavlov’s experimental work directly. Pavlov prepared several dogs on which distinct experimental procedures had been performed and he briefly explained the most important results of the experiments to his visitors. The two physiologists were impressed by the work of Pavlov. From then until 1904, both became ardent supporters of the nomination of Pavlov for the Nobel Prize in Physiology or Medicine (Todes, 2000).

Despite this positive report, the Committee’s doubts persisted, also because there were very few publications under Pavlov’s name. The report did however serve for subsequent evaluations in the following years and his name remained in contention until 1904, when Pavlov obtained four votes to one, and in 1904 he was finally awarded the Nobel Prize in Physiology or Medicine (Todes, 2002).

Pavlov went to Stockholm to receive the first Nobel Prize awarded to a Russian scientist. At the age of 55, Pavlov was at the height of his career and internationally recognized, receiving a financial incentive of 73,000 gold rubles (about U$ 36,000 at the time) that he invested in his laboratory and further research (Babkin, 1949; Fernández, 2006). Interestingly, Pavlov did not seem to give too much importance to such recognition and he certainly never referred to it during his life, not even in his short autobiography. However, it did represent an important recognition of his work and that of his colleagues, as well as for his country.

Pavlov delivered his Nobel lecture in Stockholm on December 12th, 1904. He started talking about the simple topic of bread and the fight for it, which has dominated many of the events of human life. He then described the fate of food and the process of digestion, and the results of his laboratory at St. Petersburg (the Institute of Experimental Medicine). He then stopped to “… express my deepest gratitude to all my colleagues”, before he went on to describe the technical developments that had allowed them to surgically intervene in dogs, following the correct principles of anesthesia, asepsis and the proper maintenance of these facilities. He identified two key achievements: first, the digestive glands work differently depending on the nature of the food; second, this digestive process is orchestrated by the nervous system. His important discoveries provided knowledge on how nerves stimulate the gastric glands and pancreas, and how they are involved in digestive activity (Fernández, 2006).

Although Pavlov received the Nobel Prize for his research on the physiology of the digestive glands, at that time he was already interested in “psychic secretion” and what would be later known as “conditional reflexes”. The end of his lecture was devoted to this new research topic and its importance, a psychological process that would be addressed in an essentially objective and experimental way.

The Nobel Prize brought money to Pavlov and his family, as well as worldwide fame. He was invited to join different scientific communities and became a member of the Russian Academy of Sciences in 1907. By then, he led three laboratories attracting many scientists from around the world (Todes, 2000). This was interrupted by World War I and later by the Bolshevik Revolution, although once in power, Lenin gave full support to Pavlov’s work with his famous decree that allowed him to continue his research until the end of his life.

## Cajal and the Nobel Prize

When Cajal received the Nobel Prize in 1906 he was at the peak of his international recognition: he had already received the Moscow Prize in 1900 and the Helmholtz Medal in 1905. The Helmholtz Medal was purely honorary, while the other two awards were associated with a significant financial compensation. As Cajal himself indicates, the Nobel Prize winner received some “25,000 duros” (125,000 pesetas, 20,833 €).

In *Recuerdos de mi vida: Historia de mi labor científica* (*Recollections of my life*, 1923), Cajal himself mentions that once he learned of the award of the Nobel Prize in Physiology or Medicine, he felt more fear than joy. He wondered how his foreign colleagues would react and what his adversaries would say. His apprehension was justified. He was the first Spanish scientist to receive the Nobel Prize in Physiology or Medicine (which he shared with the Italian Camilo Golgi) and as Cajal himself indicated (1923), he and Golgi were “like Siamese twins, joined by the back but looking in the opposite direction”. So the award was not exempt from controversy (Armocida and Zanobio, 2006; Fishman, 2007; Grant, 2007; Fernández-Santarén, 2008). Indeed, there was also considerable controversy in the Nobel Committee’s decision process, as it was divided between awarding a shared prize to Cajal and Golgi, and presenting it to Cajal alone (see Jones, 1999, 2011; López-Muñoz et al., 2006; De Carlos and Borrell, 2007; Mazzarello, 2007; Nieto, 2012). In the end, the committee voted that the Nobel Prize should be shared by Golgi and Cajal. Cajal was nominated for the Nobel Prize from 1901 to 1906 and thus, he competed with Pavlov for 4 years. The earlier prizes were awarded to von Behring (1901), Ross (1902), Finsen (1903), Pavlov (1904), and Koch (1905), but five nominations were submitted for Cajal in 1906, the year in which he was awarded the Nobel Prize.

In Cajal’s Nobel lecture on December 12th, 1906 (Ramón y Cajal, 1907), he presented his work defending the neuron theory, referring only to facts and inferences. The speech was accompanied by many large polychrome images that graphically presented his findings to the profane. In his lecture, as expected, he praised the work of Golgi, the father of the technique with which Cajal himself had achieved so much: “He earned my admiration and all my books contain enthusiastic acclaims to the Wise Man of Pavia’s initiatives. I was therefore entitled to expect from him an equally friendly treatment of his discourse on *La doctrine de neurons*” (Ramón y Cajal, 1923). Unfortunately, Golgi did not express himself in the same way. The day before, December 11th, he focused on dismissing the recent work of many European researchers while trying to rescue his almost forgotten theory of the diffuse nerve networks. In his lecture Golgi only cited Cajal when talking about the law of dynamic polarization and the work of the internal structure of nerve cells, completely ignoring the rest of his work, to the dismay of many European researchers who attended the ceremony (López-Piñero, 2000).

By contrast, Cajal, presented much evidence supporting his conclusions in his speech, the confirmation of his observations by others, the new technical resources developed, the advantages of the reduced silver nitrate process, the proof of Kupffer and His’s neurogenetic doctrine, the evidence obtained from the regenerative mechanism of nerves, and the evidence from embryonic neurogenesis. He ended his speech with the following words: “In short, the set of observations just outlined, and many others of which I have not had time to speak, supports His’s neurogenetic doctrine as an inevitable postulate, a doctrine formulated by that forgotten scholar whose eminent work has suffered the injustice of seeing a phalanx of young scientists describing his finest and greatest discoveries as mistakes in recent years” (Ramón y Cajal, 1907).

Cajal became immensely popular in Spain, making him a living legend. This enabled him to obtain the support of the government and crystallize important institutional projects that were to have an important impact on science and Spanish scientists until the outbreak of civil war in 1936.

## Concluding Remarks

In conclusion, Santiago Ramón y Cajal and Ivan Petrovich Pavlov were contemporary scientists and while they had a similar vision of science, they had completely opposite views on how to manage their particular schools and disciples. Pavlov exerted a hierarchical control over the work of his disciples, while Cajal offered his guidance, support and freedom for them to develop and expand their own research interests. While we do not know with certainty if they ever met in person, they certainly both attended the Congress in Madrid in 1903, and it is very likely that they were introduced to one another there. The 80th anniversary of Cajal’s death on October 17th, 2014 coincided with the expiration of the copyright the family held over his legacy. This sets a new stage for research into different aspects of Cajal’s work and personal life, and a further analysis of his personal correspondence with other scientists, such as Pavlov.

## Conflict of Interest Statement

The authors declare that the research was conducted in the absence of any commercial or financial relationships that could be construed as a potential conflict of interest.
